# Comparison between biparametric and multiparametric MRI in predicting muscle invasion by bladder cancer based on the VI-RADS

**DOI:** 10.1038/s41598-022-19273-7

**Published:** 2022-11-30

**Authors:** Tae Il Noh, Ji Sung Shim, Sung Gu Kang, Deuk Jae Sung, Jun Cheon, Ki Choon Sim, Seok Ho Kang

**Affiliations:** 1 Department of Urology, Anam Hospital, Korea University College of Medicine, 73 Goryeodae-Ro, Seongbuk-Gu, Seoul, 02841 Korea; 2 Department of Radiology, Anam Hospital, Korea University College of Medicine, Seoul, Korea

**Keywords:** Urology, Bladder

## Abstract

This study aimed to compare the diagnostic validity of biparametric magnetic resonance imaging (bpMRI) with that of multiparametric MRI (mpMRI) based on the Vesicle Imaging-Reporting and Data System (VI-RADS) in predicting muscle invasion by bladder cancer (BCa). We retrospectively examined 357 patients with an initial diagnosis of BCa who underwent preoperative MRI; 257 and 100 patients underwent mpMRI and bpMRI, respectively. Two urogenital radiologists evaluated all bpMRI and mpMRI scans using VI-RADS, and the diagnostic validity of VI-RADS for predicting muscle invasion by BCa was analyzed based on histopathology of the first and/or second transurethral resection of bladder tumors and radical cystectomy. Receiver operating characteristic (ROC) curves were plotted with the calculation of area under the curves (AUCs), and the level of significance was P < 0.05. Both groups showed optimal performance with a VI-RADS score ≥ 3. BpMRI showed comparable diagnostic performance to mpMRI (reader 1: AUC, 0.903 [0.827–0.954] vs. 0.935 [0.884–0.968], p = 0.510; and reader 2: AUC, 0.901 [0.814–0.945] vs. 0.915 [0.874–0.946]; p = 0.655). The inter-reader agreement between both readers was excellent (Cohen’s kappa value = 0.942 and 0.905 for bpMRI and mpMRI, respectively). This comparative study suggests that bpMRI has comparable diagnostic performance to mpMRI and may be an alternative option to predict muscle invasion by BCa.

## Introduction

Bladder cancer (BCa) is among the most commonly diagnosed cancers worldwide; it comprises 3% of all newly diagnosed cancers and is 3.4 times more common in men than in women^1^. BCa is classified as non-muscle invasive BCa (NMIBC) or muscle invasive BCa (MIBC) depending on whether the muscularis has been invaded^2^. Approximately 20–25% of newly diagnosed BCas are MIBCs. Moreover, approximately 70% of patients experience NMIBC recurrence even after initial treatment with transurethral resection of a bladder tumor (TURBT). Of these patients, 10–20% experience progression to MIBC^3^.

The surgical management and prognoses of NMIBC and MIBC are entirely different from each other^4^. Although the recommendations for BCas are established based on complex clinical, pathological, and imaging factors, the treatments are based on local staging primarily with specimens obtained by TURBT. For NMIBCs, TURBT with or without intravesical therapy has been used as a primary treatment to reduce recurrence and progression to MIBC^5^. Contrastingly, for MIBCs, which demonstrate the invasion of the muscularis propria layer in TURBT, radical cystectomy (RC) with lymph node dissection is the surgical standard. Even postoperatively, chemotherapy and radiotherapy are required for unfit patients undergoing RC, due to the aggressiveness and poor prognosis of MIBC^6^. Therefore, it is essential to confirm the local stage to decide treatment strategies. For all tumors diagnosed as T1 high grade (HG), as well as those not including the detrusor muscle in the specimen, a second look and repeated TURBT (re-TURBT) are recommended^5,7^.

However, since re-TURBT has a risk of complications, including bleeding and bladder wall perforation^8^, magnetic resonance imaging (MRI) without radiation is being increasingly considered for the staging of BCa as the modality of choice for imaging. The Vesical Imaging-Reporting and Data System (VI-RADS), a multiparametric MRI (mpMRI) reporting system for BCa staging and muscle invasion prediction, has recently been launched^8^. It shows high sensitivity, specificity, and accuracy for predicting the invasiveness of bladder tumors in the muscle layer^9,10^.

The guidelines of the European Association of Urology (EAU) recommend the inclusion of multiplanar T2-weighted imaging (T2WI), diffusion-weighted imaging (DWI), and dynamic contrast-enhanced (DCE) MRI in a mpMRI protocol for VI-RADS scoring^6^.

Although a contrast-free protocol is a relatively novel topic in the literature for BCa, our study was prompted by the question of whether the contrast material, considering its side effects, is truly essential in predicting muscle invasion. Only a few studies with a small sample size have compared the diagnostic performance between biparametric MRI (bpMRI) and mpMRI; both imaging modalities had similar sensitivity and specificity^11–15^. The present study aimed to validate the use of VI-RADS in patients with BCa and compare its diagnostic performance for predicting muscle invasion between two different protocols: bpMRI and mpMRI. Herein, we investigated whether contrast media is essential and whether contrast-free bpMRI without a DCE sequence can be considered an alternative to standard mpMRI.

## Materials and methods

### Study population and design

We retrospectively examined 357 patients between January 2018 and June 2021 with an initial diagnosis of BCa who underwent preoperative MRI; 257 and 100 patients underwent mpMRI and bpMRI, respectively.

Inclusion criteria were as follows: (1) patients with suspected bladder mass on computed tomography (CT) and/or cystoscopy examination; (2) patients who underwent bpMRI or mpMRI using a 3.0-T scanner; and (3) patients with subsequent TURBT-proved urothelial BCa. Exclusion criteria were as follows: (1) patients who underwent biopsy alone due to unfitness for proper TURBT (mpMRI group, n = 4; and bpMRI group, n = 1); and (2) patients who had severe susceptibility artifacts in the pelvis (i.e., hip joint replacement) (mpMRI group, n = 2).

Between January 2018 and December 2018, a predetermined number (n = 100) of patients with BCa satisfying the inclusion/exclusion criteria underwent bpMRI, which was performed before mpMRI with a DCE sequence was recommended and adopted in our institution. After the adoption of VI-RADS, 257 patients with BCa satisfying the inclusion/exclusion criteria between January 2019 and June 2021 underwent mpMRI. The endpoint of this study was to compare the diagnostic performance of mpMRI with that of bpMRI in predicting MIBC.

### mpMRI and bpMRI examinations

Patients were prepared according to the guidelines of the EAU^8^ and Sim et al.’s method^16^. All MRI examinations were performed using 3.0-T equipment (Magnetom Skyra and Prisma, Siemens Healthineers, Erlangen, Germany or Achieva, Philips Healthcare, Best, Netherlands) and a multichannel phased-array external surface coil. The entire pelvis was imaged from the aortic bifurcation to the inferior margin of the pubic symphysis. High-resolution fast spin-echo nonfat-suppressed T2-weighted images in three orthogonal planes were obtained. DWI was performed during free breathing using a water-excited single-shot spin-echo echo-planar sequence in the axial planes. DCE MR images were acquired using an axial fat-suppressed three-dimensional volumetric spoiled gradient-echo sequence before and after intravenous injection of 0.2 mL/kg of gadotarate meglumine (Dotarem^®^; Guerbet) at 2 mL/s, up to a total volume of 20 mL. Four sets of contrast-enhanced images were obtained 30–120 s after contrast material injection. The detailed MRI protocols are summarized in Supplemental Table 1.

### MR image interpretation

**Figure 1:**
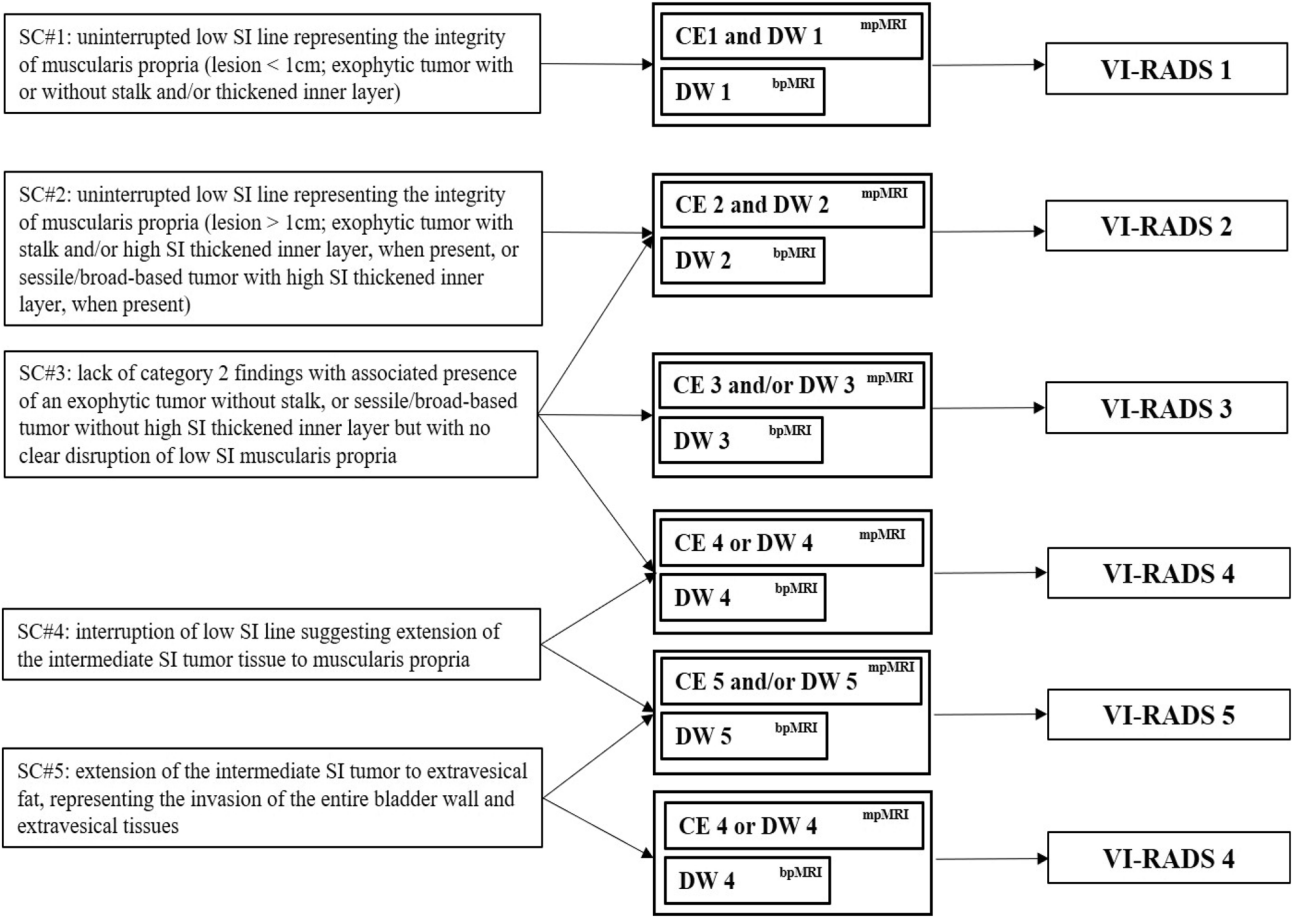
Summary of the schematic representation of the bp- and mpVI-RADS scoring interpretation. A five-point biparametric VI-RADS (bpVI-RADS) score is generated using the individual T2WI and DWI. The T2-weighted sequence is the dominant sequence, and the diffusion-weighted sequence is considered as a first pass guide. For categories 1–3, the “first pass scoring” T2 sequence should be considered. For categories 4 and 5, the dominant sequences (i.e., when image quality is optimal) are present in DWI for bpVI-RADS, DWI (first) and DCE (second; especially if the DWI is suboptimal) for mpVI-RADS. *bpMRI* biparametric MRI, *DW* diffusion-weighted category, *DWI* diffusion-weighted imaging, *mpMRI* multiparametric MRI, *SC* structural category, *SI* signal intensity, *VI-RADS* Vesicle Imaging-Reporting and Data System.

For comparison between MRI images, two urogenital radiologists with > 10 years of experience through separate sessions evaluated all bpMRI and mpMRI scans using the VI-RADS classification system (Fig. 1). The reviewers were blinded to the clinical, surgical, and histopathological results associated with the images. All lesions were scored separately according to the VI-RADS criteria using images obtained via T2WI, DWI, and/or DCE MRI. The VI-RADS score reflecting the overall risk score of detrusor muscle invasion for each patient was assigned as described in the literature according to the following findings^8^:

- T2WI: Interruption of the hypointense line of the muscle layer by a tumor with T2 intermediate signal intensity or extension of the tumor to the extravesical fat.
- DWI: Extension of a hyperintense tumor on DWI and low signal intensity on an apparent diffusion coefficient (ADC) map to the hypointense line of the muscle layer or extravesical fat.
- DCE: Extension into the hypointense line of the muscle layer by early enhancement of the tumor or the tumor stalk not being observed.

**Figure 2:**
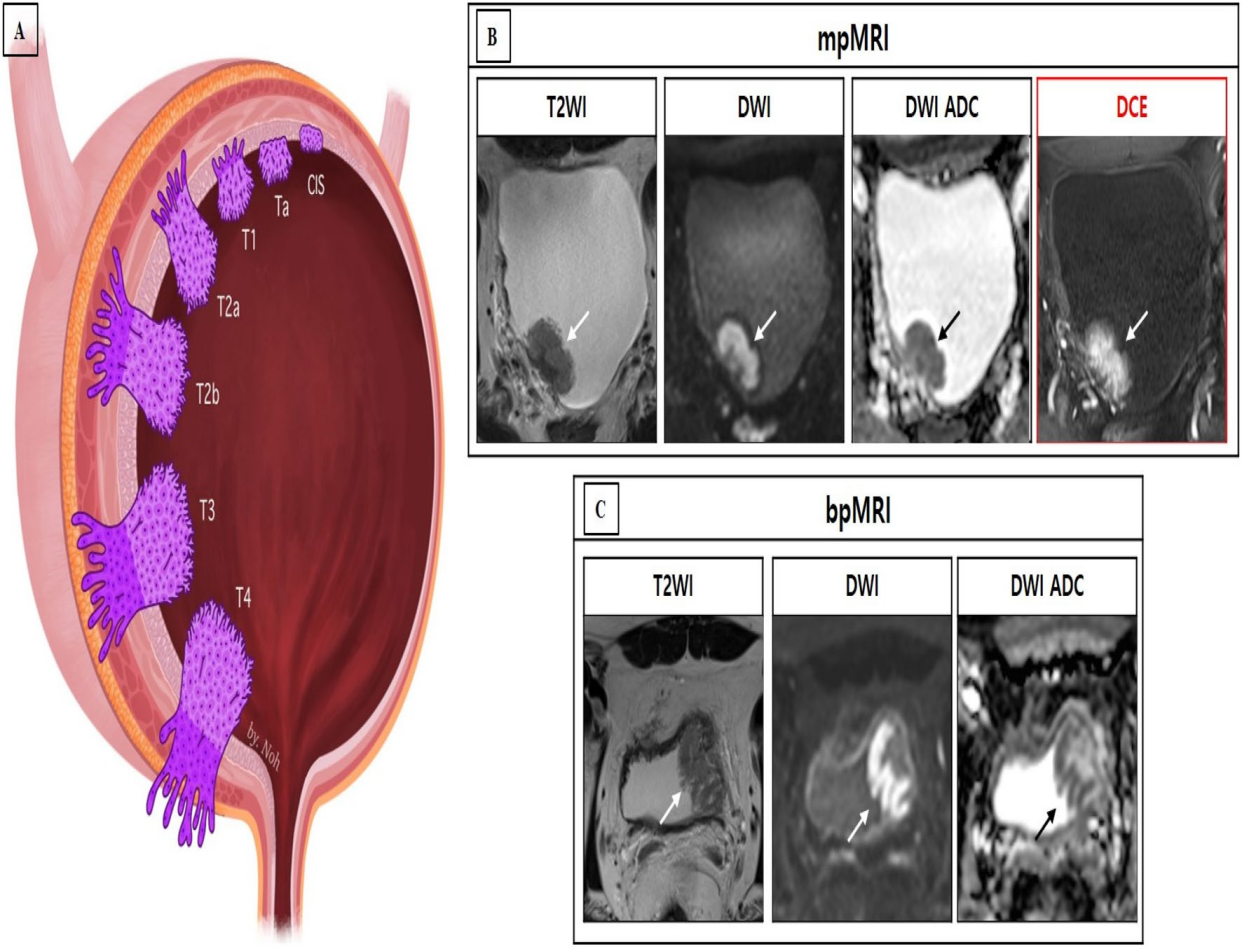
Schematic representation of the bladder cancer stage and case presentation of the VI-RADS scoring. **(A)** Bladder wall anatomy and local bladder tumor staging. (**B**) Multiparametric MRI (mpMRI) in a 63-year-old man. The bladder tumor was categorized as SC 4, DW 3, and CE 4 in axial T2-weighted magnetic resonance image (T2WI), diffusion-weighted image (DWI) with a high *b* value and apparent diffusion coefficient map (ADC), and dynamic contrast enhanced image (DCE) obtained 30 s after the administration of contrast material, respectively. The VI-RADS score was 4, and urothelial carcinoma (stage T2) was confirmed by the transurethral resection of bladder tumor (TURBT). (**C**) Biparametric MRI (bpMRI) in a 60-year-old man. The bladder tumor was categorized as SC 3 and DW 2 in axial T2WI and DWI with a high *b* value and ADC respectively. The VI-RADS was 2, and urothelial carcinoma (stage T1) was confirmed by TURBT. *ADC* apparent diffusion coefficient, *DCE* dynamic contrast enhanced, *T2WI* T2-weighted imaging, *VI-RADS* Vesicle Imaging-Reporting and Data System.

The VI-RADS scores are as follows^8^:

- VI-RADS score 1: Uninterrupted low SI line representing muscularis integrity (size, < 1.0 cm).
- VI-RADS score 2: Similar to VI-RADS score 1, except for a size > 1.0 cm and a thickened inner layer.
- VI-RADS score 3: Disappearance of category 2 findings, but no clear disruption of low SI muscularis layer.
- VI-RADS score 4: Interruption of low SI line suggesting extension into the muscularis layer.
- VI-RADS score 5: Extension of the intermediate SI tumor to extra-vesical fat.
  The schematic representation and scoring of VI-RADS for bpMRI are as follows (Figs. 1 and 2):
- VI-RADS 1 (muscle invasion is highly unlikely): SC and DW category 1.
- VI-RADS 2 (muscle invasion is unlikely): SC and DW category 2; and SC category 3 with DW category 2. In VI-RADS score 2, muscle invasion is unlikely.
- VI-RADS 3 (presence of muscle invasion is equivocal): SC and DW category 3.
- VI-RADS 4 (muscle invasion is likely): At least SC and/or DW category 4; SC category 3 with DWI category 4; and SC category 5 with DW category 4.
- VI-RADS 5 (invasion of muscle and beyond the bladder is very likely): SC and DW category 5.

### Standard of reference

TURBT was performed for all lesions of suspected BCa by a single surgeon with 15 years of experience and performed surgeries in > 200 cases of TURBTs and 50 cases of intracorporeal robot-assisted radical cystectoprostatectomy with urinary diversion annually, amassing a total of 300 cases.

Re-TURBT was performed for tumors diagnosed as T1 HG tumors; this classification does not guarantee appropriate resection of all tumors and does not include the detrusor muscle in the specimen after primary TURBT. Subsequently, RC was performed in patients diagnosed with T1 HG (Bacillus Calmette–Guerin [BCG] unresponsive), T2, or higher-grade tumors. The histopathologic results of TURBT, re-TURBT, and RC performed by a single surgeon were used as the standard reference. The specimens were evaluated for the presence of muscle invasion by a dedicated uropathologist with 10 years of experience and blinded to MRI findings during evaluation. Tumor-node-metastasis staging and tumor grade were classified according to the World Health Organization class standards^17^.

### Statistical analysis

To quantify and compare the discriminatory accuracy of each modality for identifying the muscle invasiveness of BCa, receiver operating characteristic (ROC) curve analyses were performed, and the results were summarized as the areas under the ROC curves (AUCs) and 95% confidence intervals (CIs). The VI-RADS cut-off were determined by the ROC curves analysis by Youden’s index. The sensitivity and specificity of the cut-off value of each VI-RADS score were determined using a 2 × 2 contingency table. Descriptive statistics were calculated using VI-RADS, ≥ 3 as the cut-off for muscle invasion by BCa (Supplemental Table 2). For each reader, the ROC curves were computed for bpMRI and mpMRI, and a pairwise comparison of AUCs was performed using Delong’s test. Inter-reader agreement between readers 1 and 2 was assessed using Cohen’s Kappa statistics.

All statistical analyses were performed using IBM SPSS version 24.0 (IBM Corp., Armonk, NY, USA) and R software version 3.6.1 (R Foundation for Statistical Computing, Vienna, Austria). A p-value < 0.05 was considered statistically significant.

### Ethics statement

This study was conducted according to the guidelines of the Declaration of Helsinki and current ethical guidelines. The study was reviewed and approved by the Ethics Committee and Institutional Review Board of the Korea University Anam Hospital (IRB No. 2019AN0360). Written informed consent was obtained from all study participants prior to their enrolment.

## Results

The total number of BCas analyzed was 357, with 100 patients undergoing bpMRI and 257 undergoing mpMRI. Table 1 shows the characteristics of both groups, with the bpMRI group including 84 men and 16 women aged 69.2 ± 11.5 years and the mpMRI group including 217 men and 40 women aged 68.5 ± 12.5 years. All patients underwent TURBT [bpMRI group: NMIBC, 76 (76.0%, more in detail; Tis, 2; Ta, 34; and T1:40) and MIBC, 24 (24.0%); mpMRI group: NMIBC, 188 (73.2%, more in detail; Tis, 2; Ta, 96; and T1:90) and MIBC, 69 (26.8%)].

**Table 1:** Characteristics of patients with bladder cancer lesions using VI-RADS.

Variable (%)	Multiparametric MRI						Biparametric MRI					
	Total	VI-RADS 1	VI-RADS 2	VI-RADS 3	VI-RADS 4	VI-RADS 5	Total	VI-RADS 1	VI-RADS 2	VI-RADS 3	VI-RADS 4	VI-RADS 5
Number	257	28 (10.9)	147 (57.2)	31 (12.1)	20 (7.7)	31 (12.1)	100	18 (18.0)	49 (49.0)	13 (13.0)	8 (8.0)	12 (12.0)
Age (year), mean ± SD	68.5 (12.5)						69.2 (11.5)					
**Sex**												
Male	217 (84.4)	23 (8.9)	127 (49.4)	28 (10.9)	19 (7.4)	21 (8.2)	84 (84.0)	15 (15.0)	40 (40.0)	12 (12.0)	8 (8.0)	9 (9.0)
Female	40 (15.6)	5 (1.9)	21 (8.2)	3 (1.2)	1 (0.4)	10 (3.9)	16 (16.0)	3 (3.0)	9 (9.0)	1 (1.0)	0 (0.0)	3 (3.0)
**No. of lesions**												
Unifocal	136 (52.9)	19 (7.4)	74 (28.8)	16 (6.2)	12 (4.7)	9 (3.5)	59 (59.0)	16 (16.0)	26 (26.0)	8 (8.0)	2 (2.0)	9 (9.0)
Multifocal	121 (47.1)	9 (3.5)	71 (27.6)	11 (4.3)	8 (3.1)	22 (8.6)	41 (41.0)	2 (2.0)	23 (23.0)	5 (5.0)	6 (6.0)	2 (2.0)
**Diameter of index lesion size**												
< 1 cm	28 (10.9)	28 (10.9)	0 (0.0)	0 (0.0)	0 (0.0)	0 (0.0)	18 (18.0)	18 (18.0)	0 (0.0)	0 (0.0)	0 (0.0)	0 (0.0)
1–3 cm	134 (52.1)	0 (0.0)	111 (43.2)	17 (6.6)	4 (1.6)	2 (0.8)	44 (44.0)	0 (0.0)	34 (34.0)	7 (7.0)	2 (2.0)	1 (1.0)
> 3 cm	95 (37.0)	0 (0.0)	36 (14.0)	14 (5.4)	16 (6.2)	29 (11.3)	38 (38.0)	0 (0.0)	15 (15.0)	6 (6.0)	6 (6.0)	11 (11.0)
**T stage**												
Tis	2 (0.8)	1 (0.4)	1 (0.4)	0 (0.0)	0 (0.0)	0 (0.0)	2 (2.0)	1 (1.0)	1 (1.0)	0 (0.0)	0 (0.0)	0 (0.0)
Ta	96 (37.4)	19 (7.4)	69 (26.8)	8 (3.1)	0 (0.0)	0 (0.0)	34 (34.0)	14 (14.0)	16 (16.0)	4 (4.0)	0 (0.0)	0 (0.0)
T1	90 (35.0)	8 (3.1)	66 (35.7)	11 (4.3)	5 (1.9)	0 (0.0)	40 (40.0)	3 (3.0)	28 (28.0)	6 (6.0)	3 (3.0)	0 (0.0)
≥ T2	69 (26.8)	0 (0.0)	11 (4.3)	12 (4.7)	15 (5.8)	31 (12.1)	24 (24.0)	0 (0.0)	4 (4.0)	3 (3.0)	5 (5.0)	12 (12.0)
Concomitant CIS	94 (37.0)	10 (3.9)	56 (21.8)	11 (4.3)	9 (3.5)	8 (3.1)	37 (37.0)	4 (4.0)	20 (20.0)	5 (5.0)	3 (3.0)	5 (5.0)
**Pathology grade**												
Low	46 (17.9)	10 (3.9)	29 (11.3)	7 (2.7)	0 (0.0)	0 (0.0)	22 (22.0)	9 (9.0)	10 (10.0)	3 (3.0)	0 (0.0)	0 (0.0)
High	211 (82.1)	16 (6.2)	117 (45.5)	24 (9.3)	20 (7.8)	31 (12.1)	78 (78.0)	8 (8.0)	39 (39.0)	10 (10.0)	8 (8.0)	12 (12.0)
re-TURBT	49 (54.4)	6 (75.0)	33 (50.0)	8 (72.7)	1 (20.0)	0 (0.0)	15 (37.5)	1 (33.3)	9 (32.1)	2 (33.3)	3 (100.0)	0 (0.0)
Upstage	2 (4.1)	0 (0.0)	1 (3.0)	1 (12.5)	0 (0.0)	0 (0.0)	1 (6.7)	0 (0.0)	0 (0.0)	1 (50.0)	0 (0.0)	0 (0.0)
Cystectomy	43 (16.7)	0 (0.0)	10 (3.9)	8 (3.1)	9 (3.5)	16 (6.2)	20 (20.0)	0 (0.0)	4 (4.0)	3 (3.0)	4 (4.0)	9 (9.0)
**MRI**												
Scan time (SD), min	35.3 (5.7)						24.4 (4.2)					
Cost (SD), USD	1320 (23.2)						1073(15.4)					

Percentages are presented in parentheses.

*CIS* carcinoma in situ, *VI-RADS* Vesical Imaging Reporting and Data System, *re-TURBT* repeated transurethral resection of a bladder tumor, *Tis* carcinoma in situ, *Ta* noninvasive papillary carcinoma, *T1* tumor invades subepithelial connective tissue, *T2* tumor invades muscular propria, *MRI* magnetic resonance imaging, *SD* standard deviation, *USD* United States dollar.

In the bpMRI group, 15 of 40 (37.5%) patients underwent re-TURBT, and upstaging from T1 to T2 in the re-TURBT group was confirmed in 1 of 15 patients (6.7%), who was 1 of the 8 patients with VI-RADS 3 score in the re-TURBT group. In the mpMRI group, 49 of 90 (54.4%) patients underwent re-TURBT, and upstaging from T1 to T2 in the re-TURBT group was confirmed in 2 of 49 (4.1%) patients in the re-TURBT group.

The rates of muscle invasion were 8.2% and 7.5% for VI-RADS score 2, 23.1% and 38.7% for VI-RADS score 3, 62.5% and 75.0% for VI-RADS score 4, and 100% and 100% for VI-RADS score 5 groups for bpMRI and mpMRI, respectively. RC was performed following TURBT in 20 of 100 (20.0%) cases in the bpMRI group and in 43 of 257 (16.7%) cases in the mpMRI group. The mean acquisition time was shorter in bpMRI than in mpMRI [bpMRI, 24.4 ± 4.2 min (mean ± SD); and mpMRI 35.3 ± 5.7 min (mean ± SD) (p = 0.045)]. The cost ± SD for bpMRI and mpMRI were US$1,073 (15.4) and US$1,320 (23.2), respectively.

**Figure 3:**
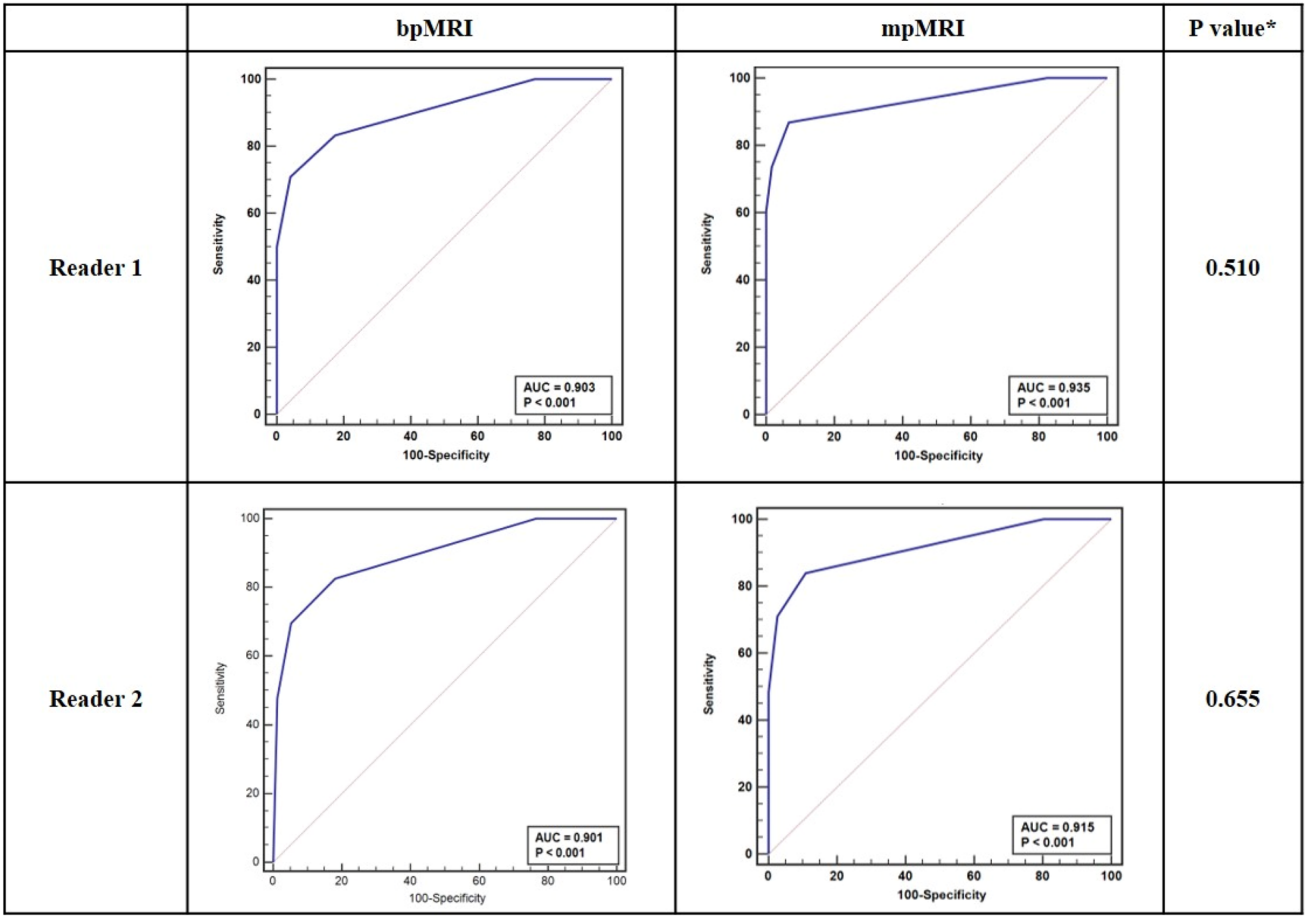
Comparison of ROC curve and inter-reader agreement between bpMRI and mpMRI. A VI-RADS score ≥ 3 was assumed to define MIBC. Asterisk: comparison of two independent ROC curves between bpMRI and mpMRI was performed using Delong’s test. *AUC* area under the curve, *bpMRI* biparametric magnetic resonance imaging, *mpMRI* multiparametric magnetic resonance imaging, *ROC* receiver operating characteristics.

BpMRI showed optimal performance with a VI-RADS score ≥ 3 as follows: reader 1, sensitivity (95% CI): 83.3% (62.6%–95.3%) and specificity: 82.4% (71.8%–90.4%); and reader 2, sensitivity (95% CI): 82.6% (61.2%–95.0%) and specificity: 81.8% (71.4%–89.7%). mpMRI also showed an optimal performance with a cut-off of VI-RADS score ≥ 3 as follows: reader 1, sensitivity (95% CI): 86.8% (71.9%–95.6%) and specificity: 93.3% (87.2%–97.1%); and reader 2, sensitivity (95% CI): 83.9% (72.3%–92.0%) and specificity: 89.1% (83.8%–93.1%). VI-RADS score ≥ 3 was assumed to define MIBC, and the ROC curves were constructed accordingly. The AUCs (95% CI) of bpMRI and mpMRI with the criterion of VI-RADS score ≥ 3 were comparable (reader 1: 0.903 [0.827–0.954] vs. 0.935 [0.884–0.968], p = 0.510; and reader 2: 0.901 (0.814–0.945) and 0.915 (0.874–0.946), p = 0.655, in bpMRI vs. mpMRI, respectively) (Table 2, Fig. 3).

**Table 2:** Diagnostic performance between both readers in predicting muscle invasion by bladder cancer.

VI-RADS ≥ 3	Reader 1			Reader 2		
	bpMRI	mpMRI	P value	bpMRI	mpMRI	P value
Sensitivity (95% CI)	83.3 (62.6–95.3)	86.8 (71.9–95.6)	0.405	82.6 (61.2–95.0)	83.9 (72.3–92.0)	0.512
Specificity (95% CI)	82.4 (71.8–90.4)	93.3 (87.2–97.1)	0.048	81.8 (71.4–89.7)	89.1 (83.8–93.1)	0.108
AUC (95% CI)	0.903 (0.827–0.954)	0.935 (0.884–0.968)	0.510	0.901 (0.814–0.945)	0.915 (0.874–0.946)	0.655

*AUC* area under the curve, *bpMRI* biparametric magnetic resonance imaging, *mpMRI* multiparametric magnetic resonance imaging, *VI-RADS* Vesical Imaging Reporting and Data System, *CI* confidence interval.

There was excellent inter-reader agreement between readers 1 and 2 in the VI-RADS scores using bpMRI and mpMRI, and the agreement value (Cohen’s kappa value) of both readers was 0.942 and 0.905 for bpMRI and mpMRI, respectively.

## Discussion

The treatment method for BCa can be determined based on complex factors such as stage, tumor grade, subtype of histopathology, and response to intravesical therapies such as BCG and chemotherapeutic agents^5^. However, one of the primary considerations regarding decisions for the prognosis and management of BCa is the local tumor stage. Although BCa staging is established based on a combination of clinical, pathological, and imaging methods, the local tumor stage is primarily determined according to the presence or absence of muscle invasion based on pathological specimens obtained by TURBT^6^.

The main framework of treatment is completely different depending on the presence or absence of muscle invasion. Therefore, predicting muscle invasion is essential in determining treatment; re-TURBT is recommended for confirming the presence of muscle invasion for all T1 HG tumors, as well as tumors in which no muscularis propria is included in the specimen^7^.

Primary TURBT may be inadequate, with a high percentage of residual tumors (33%–76%) and a high upstaging rate (0%–32%) after re-TURBT. Re-TURBT has been recommended as a second-look procedure for residual tumors and the confirmation of the presence of the muscularis propria; however, re-TURBT has a risk of possible complications such as perforation and bleeding^18^.

To reduce the potential risks of re-TURBT, radiologic imaging has been proposed to discriminate MIBCs from NMIBCs. Both CT and mpMRI are available for the diagnosis and assessment of stages of local BCa before TURBT according to the guidelines of the EAU^6^. For the detection and staging of BCa, CT urography is generally performed. However, this method has its own drawbacks in predicting muscle invasion by BCa^19^. To distinguish MIBCs from NMIBCs, VI-RADS with MRI was developed, and published literature has demonstrated its clinical reliability^20–22^. With the guarantee of appropriate tumor resection by an experienced surgeon and no suspicion of muscle invasion on MRI with VI-RADS, re-TURBT could be omitted and avoided^23^.

Furthermore, MRI based on VI-RADS has the perspective applicability to be broadly used for follow-up in patients with BCa^23^. Two potential applications of MRI based on VI-RADS are assessment of responses to systematic therapy and risk assessment^24^ and active surveillance. Active surveillance may be considered in patients with low risk NMIBC^25^. To reduce the potential risk of surgical management and discomfort from frequent cystoscopy following TURBT, an alternative tool using a regression algorithm (Xpert Bladder Monitor Test) for active surveillance has also been reported^26^. As T2WI MRI provides optimal soft-tissue contrast, mpMRI for the standardized discrimination of MIBCs from NMIBCs and its potential applications are promising. However, some possible drawbacks, such as mpMRI being time consuming and costly^27^ and the potential risks associated with the contrast media, such as contrast-induced nephropathy including nephrogenic systemic fibrosis, renal insufficiency, renal failure, and the accumulation of gadolinium, should be considered^28,29^. The incidence rate of contrast media-induced nephropathy ranged from 0.15%–2.0%, is related to an increase of ≥ 25% in serum creatinine levels, and is responsible for approximately 10% of cases of hospital-acquired acute renal failure in Europe and the United States^30,31^. To overcome these drawbacks of mpMRI, several alternative imaging tools have been proposed. One noteworthy imaging tool is the high-resolution microultrasound imaging proposed for BCa detection and staging^32^, which shows an excellent performance compared to that of mpMRI^33^. However, as high-resolution microultrasound imaging is still limited owing to the lack of equipment availability and expertise necessary, a significant learning curve related to the use of high-resolution microultrasound techniques is required^33^. Therefore, we focused on bpMRI, a contrast-free protocol based on the standardized reporting system (VI-RADS), and compared the diagnostic performance between bpMRI and mpMRI. In this study, the protocols of both bpMRI and mpMRI showed high performance, with an AUC of over 0.90. Moreover, VI-RADS has been demonstrated to be a promising predictor of muscle invasion with good reproducibility. While retaining sufficient diagnostic performance, bpMRI showed superiority in the time and cost analyses compared to mpMRI.

The value of a contrast-free protocol in BCa is a relatively novel topic; however, such a protocol has been investigated in prostate cancer based on the Prostate Imaging Reporting & Data System score, and bpMRI has shown a diagnostic performance comparable to that of mpMRI for clinically significant prostate cancer detection^34,35^.

In a study with 54 patients with BCa**,** bpMRI and mpMRI had comparable diagnostic accuracy in predicting MIBC, with no statistically significant differences in AUCs [reader 1: 0.985 (0.912–0.998) vs. 0.968 (0.884–0.996), p = 0.154; reader 2: 0.942 (0.846–0.986) and 0.936 (0.839–0.983), p = 0.345; and reader 3: 0.945 (0.848–0.986) and 0.935 (0.838–0.983), p = 0.338, in bpMRI vs. mpMRI, respectively]^13^. Moreover, the inter-reader agreement of VI-RADS was good for both bpMRI (k range, 0.814–0.86) and mpMRI (k range, 0.787–0.859)^13^. Furthermore, in a meta-analysis of 10 studies on mpMRI and 7 studies on mpMRI/bpMRI comprising ≥ VI-RADS 3 as the cut-off, the sensitivities of bpMRI and mpMRI were not significantly different (p = 0.15), yet bpMRI was more specific [0.90 (0.81–0.95) vs. 0.86 (0.77–0.91), p = 0.02, in bpMRI vs. mpMRI]^36^.

Non-contrast-enhanced MRI with only a fused high b-value DWI/ADC map and T2WI may be a promising method for assessing the depth of invasion in patients with BCa^37^. Accordingly, the issues regarding the contrast-free protocol comprise several questions regarding the major role of contrast media. It is necessary to look at each of the major roles of T2WI, DWI, and DCE sequences in predicting muscle invasion.

T2WI provides optimal soft-tissue contrast. On T2WI, the hypointense band of the muscularis propria layer is well distinguished against a marked hyperintense signal of urine and perivesical fat, including an intermediate intensity signal of the bladder tumor. Therefore, T2WI could be a key sequence for predicting muscle invasion^16^. DWI has inherent drawbacks of a low signal-to-noise ratio and spatial resolution^38^. Thus, DWI is vulnerable to disturbance by various artifacts, and its role is limited for the evaluation of small tumors and differentiation of tumors from benign conditions. To compensate for these inherent drawbacks of DWI, DCE may be considered to ensure the proper interpretation of the lesion. On a DCE sequence, tumors show rapid enhancement by contrast media, which could help distinguish tumors from the muscularis propria^39^. DCE may increase accuracy and reduce false negatives more than DWI alone. Moreover, the diagnostic performance of radiologists is not significantly affected by DCE^36^. However, DCE imaging in addition to DWI has been reported to induce a worse diagnostic performance in less experienced radiologists^14^.

Urologists establish treatment strategies for BCa based on a combination of clinical, pathological, and imaging factors through multi-modal approaches, including MRI and TURBT^5^. MRI is useful as an adjuvant tool but cannot replace tumor grading defined by TURBT^23^. Furthermore, in this study, bpMRI showed a comparable performance to mpMRI in predicting muscle invasion by BCa. Therefore, the need to add a DCE sequence to predict MIBC, with the potential risk of contrast media, is limited and uncertain.

The contrast-free protocol of bpMRI reduces the potential risks associated with intravenous contrast media. The preservation of renal function may be related to the prognosis of BCa for several reasons. First, the median age at first diagnosis is approximately 70 years, with a relatively high risk of underlying renal disease and decreased renal function^40^. Second, cisplatin-based chemotherapy, which requires eligible renal function (≥ 60 mL/min), is recommended as a basic chemotherapeutic treatment for MIBC^6^. Third, renal function can be compromised by the aggressive nature of bladder tumors, including conditions such as hydronephrosis and obstructive nephropathy^41^. Furthermore, the purpose of MRI for BCa is to predict MIBCs and follow the response to neoadjuvant or adjuvant chemotherapy; however, repeated intravenous contrast injection can affect renal function. Thus, the preservation of renal function should be considered in the management of BCa. Other benefits of avoiding DCE with contrast media include a shorter MRI scan time, improved patient comfort, no worries of contrast allergy, and reduced cost^42^.

The results of the present study demonstrate that VI-RADS is reliable and reproducible for predicting muscle invasiveness, regardless of the MRI protocol. Furthermore, bpMRI is more advantageous than the contrast-enhanced protocol, all while retaining diagnostic performance despite the absence of DCE sequences. Therefore, bpMRI could be a cost-effective alternative for predicting the muscle invasiveness of BCa.

However, to the best of our knowledge, only a few studies have compared DWI and DCE sequences for VI-RADS assessment, and most had a small sample size^11–15^. Furthermore, the authors of previous studies only compared the performance of DWI and DCE in the same patients who underwent mpMRI, not contrast-free bpMRI. DWI and DCE were separated from the same mpMRI images, and VIRADS scoring was performed based on reference to each separated sequence. Two sets of images from the same BCa patients, set 1 (T2 plus DWI) also showed comparable high performance to set 2 (T2 plus DWI plus DCE) (AUC ranged between 0.90 and 0.95 and 0.87–0.95 for bpMRI and mpMRI, P > 0.05)^11,14^. In a comparison study between bpMRI and mpMRI comprising 176 different patients (mpMRI, n = 97; and bpMRI, n = 79), the AUCs of VI-RADS using bpMRI and mpMRI were 0.88 and 0.84, respectively^15^. However, in meta-regression analyses, the number of patients (> 205 vs. ≤ 205), magnetic field strength (3.0 vs 1.5 T), and VI-RADS cut-off score (≥ 3 vs. ≥ 4) were significant factors affecting performance (p ≤ 0.03)^9^.

Therefore, the strengths of this study were the comparison between bpMRI and mpMRI with 3.0-T equipment and VI-RADS cut-off score ≥ 3, rather than a comparison between DWI and DCE sequences in the same patient. Moreover, the current study comprises an appropriately large sample size (n = 357) with excellent inter-reader agreement between two expert urogenital radiologists. Nevertheless, this study has the inherent limitations of its retrospective study design related to the heterogeneity of enrolled patients, as well as the selection and attrition biases. Therefore, well-designed randomized trials are needed to establish the utilization of bpMRI for predicting the muscle invasiveness of BCa.

## Conclusions

The current comparison between bpMRI and mpMRI in predicting muscle invasion by BCa suggests that bpMRI has a comparable performance to mpMRI while retaining the advantages of a contrast-free protocol. Therefore, bpMRI may be a cost-effective alternative for predicting the muscle invasiveness due to BCa.

## Supplementary Information

Supplementary Table S1.

Supplementary Table S2.

## Data Availability

All data generated or analyzed during this study are included in this article and its supplementary information files. The datasets used and/or analyzed in this study are available from the corresponding author upon reasonable request.

## Abbreviations

ADC: Apparent diffusion coefficient
AUC: Area under the ROC curve
BCa: Bladder cancer
BP: Biparametric
bpMRI: Biparametric MRI
CM: Contrast media
DCE: Dynamic contrast enhanced
DW: Diffusion-weighted category
DWI: Diffusion weighed imaging
HG: High grade
MIBC: Muscle invasive bladder cancer
MP: Multiparametric
mpMRI: Multiparametric MRI
MRI: Magnetic resonance imaging
NMIBC: Non-muscle invasive bladder cancer
RC: Radical cystectomy
ROC: Receiver-operating characteristic
SC: Structural category
SI: Signal intensity
TURBT: Transurethral resection of bladder tumor
T2WI: T2-weighted imaging
VI-RADS: Vesicle Imaging-Reporting and Data System
