# Supplementary material for: Comparison between biparametric and multiparametric MRI in predicting muscle invasion by bladder cancer based on the VI-RADS
Source: Sci Rep. 2022 Nov 30;12:20689. doi: 10.1038/s41598-022-19273-7 (PMC9712519; doi:10.1038/s41598-022-19273-7)
Supplement: Supplementary file 1 — Supplementary Table S1. [file 41598_2022_19273_MOESM1_ESM.docx]

Supplemental Table 1. Parameters of the T2-weighted, DWI, and DCE sequences included in the study protocol

| Parameters | T2WI | DWI | DCE (if performed) |
| --- | --- | --- | --- |
| Sequence | Fast spin echo | Single-shot EPI | Gradient echo |
| TR (msec) | 3200–3600 | 4800–7600 | 4 |
| TE (msec) | 84–97 | 54–62 | 2 |
| Flip angle (degree) | 140 | 90 | 12 |
| FOV (mm) | 230x230 or 300x300 | 300x256 | 370x301 |
| Matrix | 448x314-403 | 128x128 or 128x108 | 384x187 |
| Slice thickness (mm) | 4 | 4 | 3 |
| Slice gap (mm) | 1–1.6 | 1–1.6 | 0 |
| Number of excitations | 1 | 3 ~ 7 | 1 |
| B value | ㅡ | 0, 600, and 1000s/mm^2^ | ㅡ |

T2WI, T2-weighted imaging; DWI, diffusion-weighted imaging; DCE, dynamic contrast enhancement; EPI, echo planar imaging; TR, repetition time; TE, echo time; FOV, field of view
