# Supplementary material for: Comparison between biparametric and multiparametric MRI in predicting muscle invasion by bladder cancer based on the VI-RADS
Source: Sci Rep. 2022 Nov 30;12:20689. doi: 10.1038/s41598-022-19273-7 (PMC9712519; doi:10.1038/s41598-022-19273-7)
Supplement: Supplementary file 2 — Supplementary Table S2. [file 41598_2022_19273_MOESM2_ESM.docx]

Supplementary Table 2. Comparison of diagnostic performance between mpMRI and bpMRI based on the threshold of VI-RADS

|  |  | Reader 1 | | Reader 2 | |
| --- | --- | --- | --- | --- | --- |
| Criterion | **Protocol of MRI** | Sensitivity (95% CI) | Specificity (95% CI) | Sensitivity (95% CI) | Specificity (95% CI) |
| ≥2 | bpMRI | 100.0 (85.8–100.0) | 22.97 (14.0–34.2) | 100.0 (85.2–100.0) | 23.4 (14.5–34.4) |
|  | mpMRI | 100.0 (90.7–100.0) | 17.7 (11.3–25.7) | 100.0 (94.2–100.0) | 19.7 (14.3–26.0) |
| ≥3 | bpMRI | 83.3 (62.6–95.3) | 82.4 (71.8–90.4) | 82.6 (61.2–95.0) | 81.8 (71.4–89.7) |
|  | mpMRI | 86.8 (71.9–95.6) | 93.3 (87.2–97.1) | 83.9 (72.3–92.0) | 89.1 (83.8–93.1) |
| ≥4 | bpMRI | 70.8 (48.9–87.4) | 96.0 (88.6–99.2) | 69.6 (47.1–86.8) | 94.8 (87.2–98.6) |
|  | mpMRI | 73.7 (56.9–86.6) | 98.3 (94.1–99.8) | 70.9 (58.1–81.8) | 97.4 (94.1–99.2) |

bpMRI, biparametric magnetic resonance imaging; CI, confidence interval; mpMRI, multiparametric magnetic resonance imaging; VI-RADS, Vesical Imaging Reporting and Data System
